# Facile Synthesis of Cadmium-Free Zn-In-S:Ag/ZnS Nanocrystals for Bio-Imaging

**DOI:** 10.1038/srep24459

**Published:** 2016-04-14

**Authors:** Tong-Tong Xuan, Jia-Qing Liu, Cai-Yan Yu, Rong-Jun Xie, Hui-Li Li

**Affiliations:** 1 Engineering Research Center for Nanophotonics & Advanced Instrument, Ministry of Education, School of Physics and Materials Science, East China Normal University, Shanghai, 200062, China; 2 Sialon Group, National Institute for Materials Science (NIMS), Namiki 1-1, Tsukuba, Ibaraki 305-0044, Japan

## Abstract

High quality cadmium-free Zn-In-S:Ag doped-nanocrystals (d-NCs) were synthesized via a simple one-step noninjection route using silver nitrate, indium acetate, zinc acetate, oleylamine, S powder and 1-dodecanethiol as starting materials in an organic phase. The size and optical properties can be effectively tailored by controlling the reaction time, reaction temperature, Ag^+^ dopant concentration, and the molar ratio of In to Zn. The photoluminescence wavelength of as-prepared Zn-In-S:Ag NCs covered a broad visible range from 458 nm to 603 nm. After being passivated by protective ZnS shell, the photoluminescence quantum yield (PLQY) of Zn-In-S:Ag^+^ /ZnS was greatly improved to 43.5%. More importantly, the initial high PLQY of the obtained core/shell d-NCs in organic media can be preserved when being transferred into the aqueous media via ligand exchange. Finally, high quality Zn-In-S:Ag^+^ /ZnS d-NCs in aqueous phase were applied as bio-imaging agents for identifying living KB cells.

Doped semiconductor nanocrystals (d-NCs) have been widely investigated due to their unique optical properties^1^,^2^. They not only retain advantages of intrinsic NCs but also possess new properties, such as improved thermal and photochemical stability, longer lifetimes, and larger Stokes shift, which lead to their potential applications in solar cells, biomedical sensing, light-emitting diodes (LEDs), and so on^3^,^4^,^5^,^6^,^7^,^8^,^9^,^10^. Among them, Mn^2+^, Ag^+^, Cu^+^, and Cu^2+^ ions are often used as luminescent centers and have been demonstrated to be efficient emitters covering the blue to red color window^6^,^9^,^11^,^12^,^13^,^14^,^15^,^16^. Recently, due to the high cytotoxicity of cadmium-containing d-NCs, more attention has been paid to low toxic or toxicity-free d-NCs, such as ZnS:Cu^2+^, ZnS:Ag^+^, ZnS:Mn^2+^ , InP:Cu^2+^, and ZnSe:Cu^2+^ etc.^12^,^17^,^18^,^19^,^20^,^21^,^22^,^23^. However, the emission wavelength of these d-NCs shows poor tunability^12^,^22^,^24^. Moreover, the complex synthesis process and expensive phosphine precursor are needed upon preparation of InP based NCs^18^,^25^,^26^. For Mn^2+^ d-NCs, the dopant emission is restricted to the orange-red region because the emission comes from the intrinsic ^4^T_1_–^6^A_1_ transition of Mn^2+^ which is relatively independent of the nature and size of host NCs^12^,^14^,^27^,^28^,^29^. As to Cu or Ag d-NCs, the dopant emission can be tuned by simply controlling the size of host NCs, resulting in a narrow and tunable emission window from blue to green^6^,^9^,^11^,^30^,^31^,^32^.

Therefore, in order to achieve a much wider and tunable emission window in d-NCs, ternary I/II-III-VI compounds are promising alternatives to binary ones, because they display composition-controllable electronic and optical properties^33^,^34^,^35^,^36^,^37^,^38^. Li *et al.* reported Cu doped Zn-In-Se d-NCs with the emission covering from 545 to 620 nm, synthesized by using a hot-injection route^36^. This route usually involves complex manipulations which limit its application in a large-scale production and the control of size, due to the difficulties in controlling the rate of precursor injection, batch transfer in a short time and the reaction temperature^1^,^39^. Further, Zhong *et al.* fabricated Cu doped Zn-In-S d-NCs with the photoluminescence (PL) emission spanning over an entire visible spectral window and extending to the near-infrared spectral window, by using a noninjection approach. Using them as an active layer, the as-prepared d-NCs were successfully applied in LED devices due to the excellent PL, broad tunable emissions, and low toxicity^31^,^34^. On the other hand, the emission of Cu dopants exhibits an extremely weak stability in the open air due to the inevitable oxidation of mercapto-ligand by Cu ions^40^. By contrast, Ag d-NCs have a good stability because there is no oxidation of Ag^+^ by the mercapto-ligand. As far as stability is concerned, Ag is much better than Cu for d-NCs. With this in mind, Wang and his co-workers prepared Ag^+^ doped ZnInSe NCs by a complicated hot-injection process. The synthesized NCs had a tunable emission ranging from 504 to 585 nm, and were demonstrated their applications in bio-imaging. The problem of Ag^+^ doped ZnInSe NCs was their low photoluminescence quantum yield (PLQY, ~15%)^40^. Therefore, it is strongly desirable to develop highly efficient and wider emission window cadmium-free d-NCs to meet versatile applications such as bio-imaging and solid state lighting.

Compared with ternary chalcogenide materials like Zn-In-Se and Cu-In-S, Zn−In−S is a near-ideal candidate to serve as a host because of its high chemical stability, composition-tunable optical band gaps, low toxicity, and well-developed synthetic methods^41^,^42^,^43^. The Zn-Ag-In-S quaternary solid solution NCs have been synthesized either by thermal decomposition of a metal ion-diethyldithiocarbamate complex or the hot-injection process^44^,^45^,^46^. Both methods lead to a PLQY of 24–30%. However, to the best of our knowledge, there are no investigations on applications of these NCs.

Herein we report the synthesis of high quality Ag^+^ doped Zn-In-S d-NCs by a simple one-step noninjection synthetic method in an organic phase. The crystalline size and optical properties of the prepared d-NCs can be tailored by controlling the reaction time, reaction temperature, Ag^+^ concentration, and the molar ratio of In to Zn. After being passivated by protective ZnS shell, the as-prepared Zn-In-S:Ag^+^ /ZnS exhibit a remarkably enhanced PLQY as high as 43.5%. More importantly, the initial high PLQY of the obtained core/shell d-NCs in an organic media can be preserved when transferred into an aqueous media *via* the ligand exchange. Finally, we demonstrate that Zn-In-S:Ag^+^/ZnS d-NCs in the aqueous phase can be utilized as bio-imaging agents for identifying living KB cells.

## Results and Discussion

**Figure 1:**
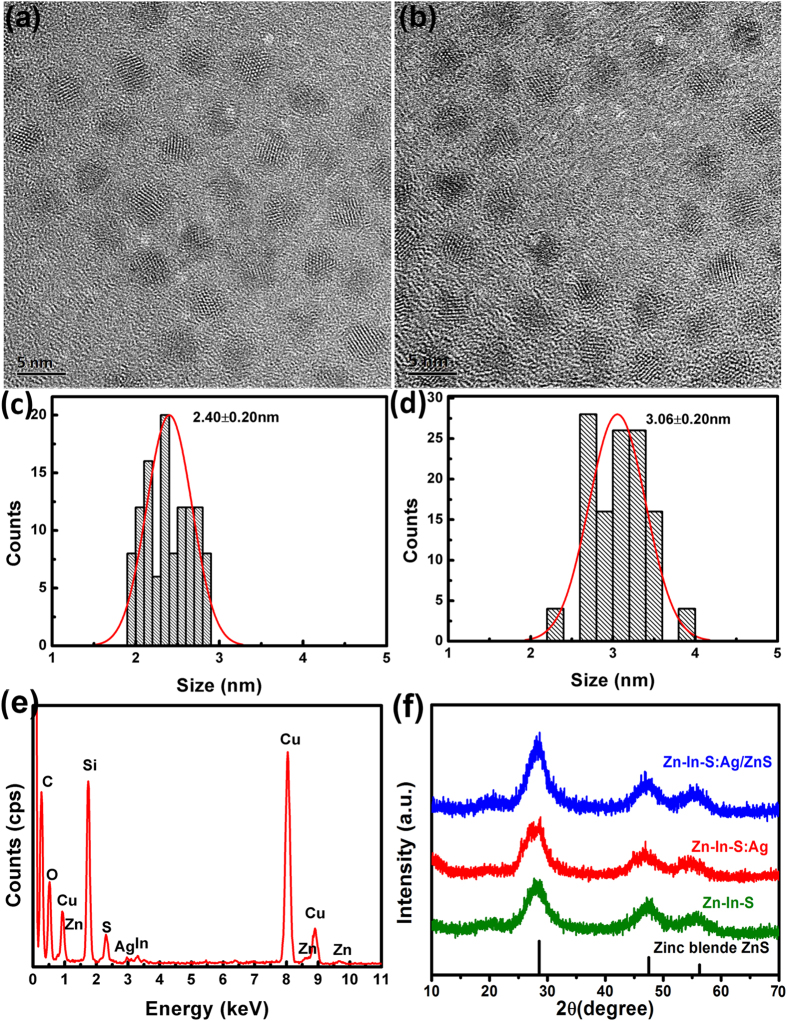
The TEM images (**a**,**b**), size distribution (**c**,**d**), and the EDS (**e**) of Zn-In-S:Ag (**a**,**c**,**e**) and Zn-In-S:Ag/ZnS d-NCs (**b**,**d**), respectively. XRD patterns (**f**) of Zn-In-S NCs, Zn-In-S:Ag d-NCs, and Zn-In-S:Ag/ZnS core/shell d-NCs.

Figures 1a–d show the typical transmission electron microscopy (TEM) photos of Zn-In-S:Ag d-NCs and Zn-In-S:Ag/ZnS core/shell d-NCs. The d-NCs exhibit monodispersity and a nearly spherical shape with a narrow size distribution. The average crystalline size of the as-prepared Zn-In-S:Ag, and Zn-In-S:Ag/ZnS d-NCs is measured as 2.40 and 3.06 nm, respectively. The energy-dispersive X-ray spectroscopy (EDS) analysis in Fig. 1e confirms the existence of Ag ions in Zn-In-S NCs. The X-ray diffraction (XRD) results (Fig. 1f) exhibit that the as-prepared Zn-In-S NCs, Zn-In-S:Ag d-NCs, and Zn-In-S:Ag/ZnS core/shell d-NCs belong to the face-centered cubic phase with the zinc blende structure (JCPDS. NO. 77-2100). The Ag incorporation causes no significant changes in the crystal structure of the Zn-In-S host NCs. Subsequently, the growth of the ZnS shell leads to a slight shift of the diffraction peaks to ZnS because of the lattice shrinkage caused by a smaller ion radii of Zn^2+^ (0.75 Å) in comparison with that of In^3+^ (0.94 Å).

**Figure 2:**
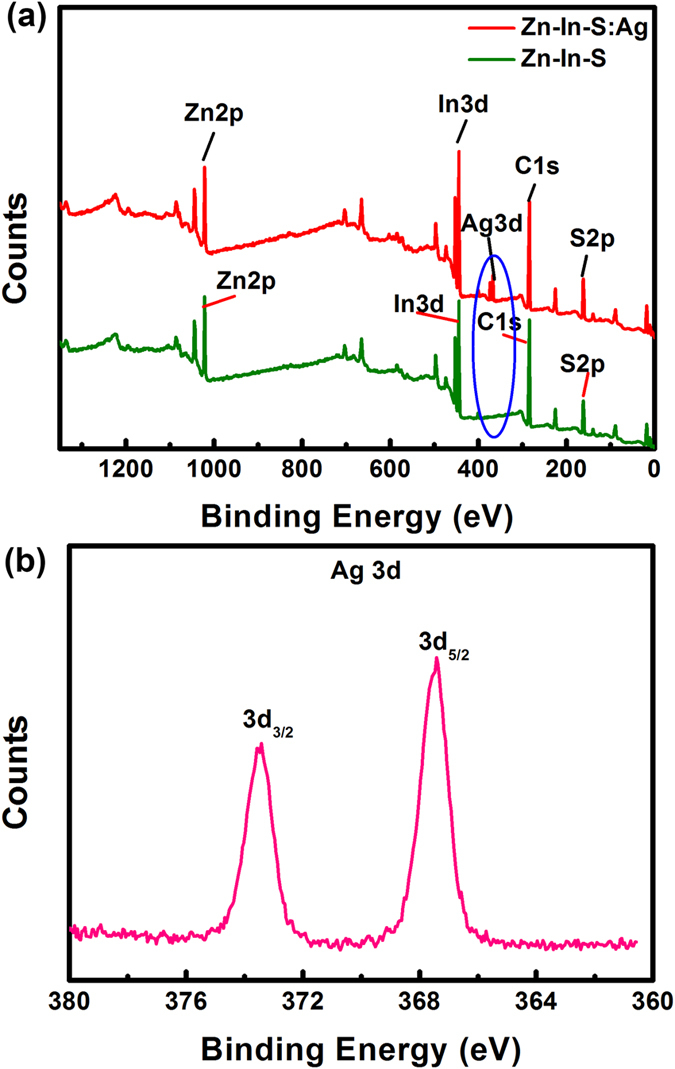
The XPS spectra of Zn-In-S NCs and Zn-In-S:Ag d-NCs (**a**). Magnification of Ag 3d peaks for Zn-In-S:Ag d-NCs (**b**).

To further confirm the Ag doping into Zn-In-S NCs, the X-ray photoelectron spectroscopy (XPS) analysis of the constituent elements was carried out. As shown in Fig. 2a, almost identical Zn 2p, In 3d, C 1 s, and S 2p XPS spectra are observed for both NCs, whereas the XPS spectrum of Ag doped Zn-In-S d-NCs exhibits two additional Ag 3d peaks, indicating that Ag ions are accommodated into the Zn-In-S NCs. The binding energies located at 367.9 eV (3d_5/2_) and 373.4 eV (3d_3/2_) (Fig. 2b) match well with the 3d signals of Ag^+^ in Ag_2_S, implying that the oxidation state of the Ag element in the Zn-In-S:Ag d-NCs is +1^40^,^47^. The signals of C 1 s in Fig. 2a should be originated from organics wrapped on the NCs surface.

**Figure 3:**
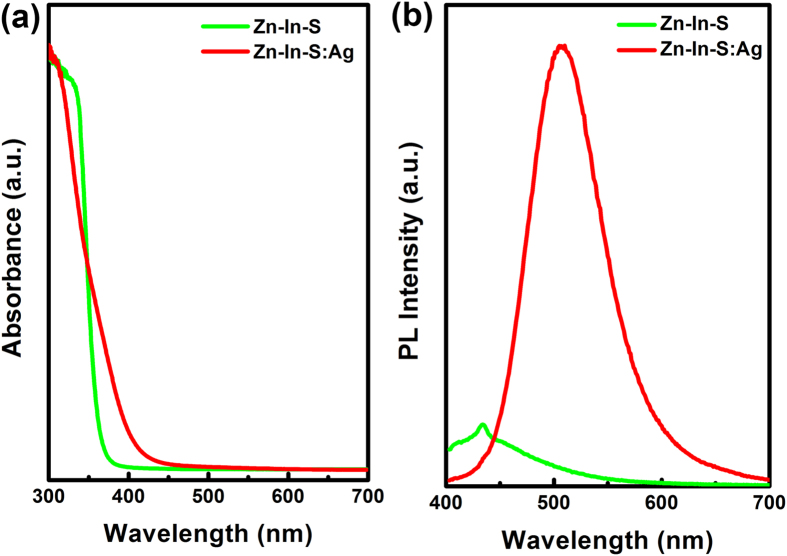
The UV/vis absorbance (**a**) and PL (**b**) spectra of NCs.

Figure 3 displays the typical UV/vis absorbance and PL spectra of Zn-In-S NCs and Zn-In-S:Ag^+^ d-NCs synthesized under the same reaction conditions. As seen, the absorption spectra of both Zn-In-S NCs and Zn-In-S:Ag^+^ d-NCs are similar (Fig. 3a). They do not show well-defined exciton absorption peaks, which is ascribed to their special electronic properties in comparison to II-VI semiconductor nanocrystals and the irregular composition distribution among different NCs. The similar absorption spectra also imply that the incorporation of Ag ions does not obviously have an influence in the particle size of NCs. On the other hand, Zn-In-S:Ag^+^ has its emission peak of 510 nm to be redshifted by 76 nm when compared to that of 434 nm for the host Zn-In-S NCs. Furthermore, the PL intensity of Zn-In-S:Ag^+^ d-NCs is also enhanced dramatically (Fig. 3b). This should only relate to the Ag dopant as a luminescent center.

**Figure 4:**
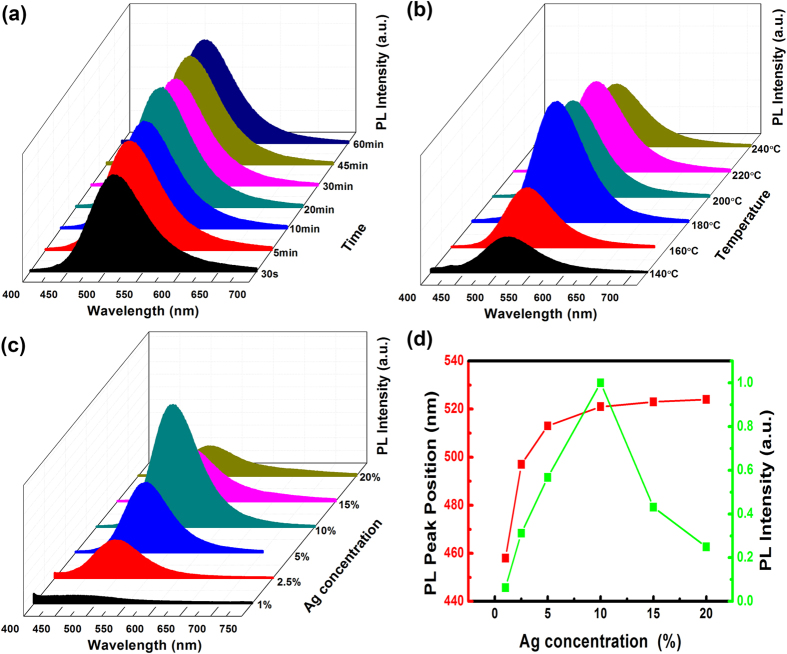
The PL spectra of Zn-In-S:Ag d-NCs. (**a**) Effect of the reaction time (temperature: 180 °C), (**b**) effect of the reaction temperature (reaction time: 20 min) (**c**) effect of the Ag doping concentration (temperature: 180 °C, reaction time: 20 min), (**d**) plots of PL intensity and the emission peak position versus the Ag concentration.

The optical properties of Zn-In-S:Ag d-NCs were optimized by investigating the synthetic conditions, and the absorption and PL spectra are illustrated in Figures S1 and 4a–b, respectively. Similar to the reported results^31^,^35^,^36^,^40^,^48^, the absorption spectra apparently shift to the longer wavelength with increasing both the reaction temperature and the reaction time, duo to the continuous growth of d-NCs based on the thermodynamics and kinetics. The reaction mobility of the ligand and precursors is enhanced when the reaction temperature is raised and the reaction time is extended, which accelerates the growth of Zn-In-S:Ag d-NCs and finally leads to the redshift in absorption. Differing from absorption spectra, the emission spectra only show a very slight redshift but a significant enhancement of the PL intensity (Fig. 4a,b), when the reaction time and temperature rise from 0.5 to 20 min and 140 to 180 °C, respectively. This is attributed to the fact that more Ag dopants adsorbed on the surface of the NCs access inside of the d-NCs. In addition, the decrease of the surface defect associated with the longer reaction time and higher temperature may also account for the increased PL intensity. As a result, the highest PL intensity of d-NCs is obtained at 180 °C for 20 min, and the PLQY is about 33.4%. Above 180 °C, the PL intensity starts to decrease, owing to the occurrence of Ostwald ripening at high temperature.

The dopant concentration of the Ag precursor is another key factor for tuning optical properties of d-NCs. As shown in Fig. 4c,d, the Ag^+^ emission exhibits a significant redshift by 66 nm (i.e., from 458 to 524 nm) and an enhancement in PL intensity as the Ag concentration increases. It results from the increased concentration of Ag ions incorporating into host NCs, which provides more holes to recombine with electrons from the bottom of the conduction band. The optimal concentration of Ag^+^ is about 10%, leading to a PLQY of 33.4%. Above 10%, the concentration quenching occurs due to the nonradiative transition between the neighboring dopant ions.

**Figure 5:**
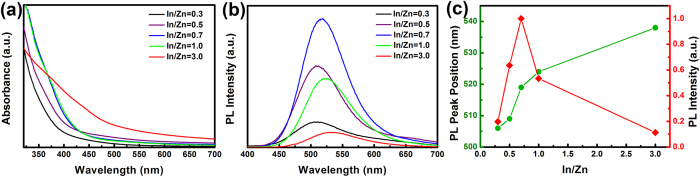
The UV/vis absorbance (**a**), PL spectra (**b**), PL peak position and intensity (**c**) of Zn-In-S:Ag d-NCs with different In/Zn ratios synthesized at 180 °C for 20 min.

**Figure 6:**
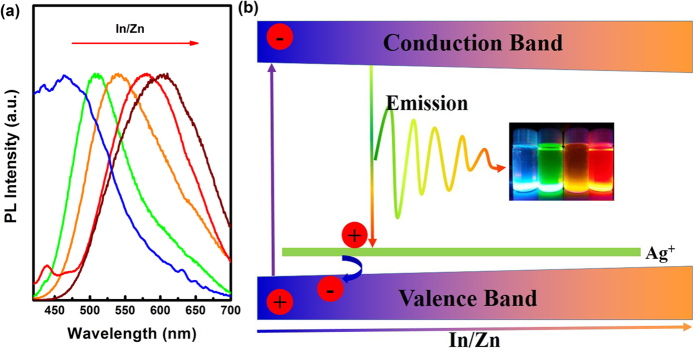
The PL spectra of Zn-In-S:Ag d-NCs with the increasing of In/Zn (**a**). The schematic representation of Ag^+^ energy state in the composition variable alloyed NCs and the possible recombination mechanism (**b**). The insert gives the photograph of Zn-In-S:Ag d-NCs with different emission wavelength under 365 nm UV light.

Similar to ternary Cu-In-S NCs^49^,^50^,^51^,^52^, the band gap and emission color of Zn-In-S:Ag d-NCs can also be tuned by varying the composition of host NCs, e.g., the ratio of In to Zn. In this work, the molar ratio of In/Zn precursor varies from 0.3 to 3.0, and the gross amount of In and Zn precursors is fixed at 0.4 mmol. As seen in Fig. 5, the onset of absorption spectra and the emission peak position are strongly related to the ratio of In/Zn (Fig. 5a,b). With the increase of the nominal In/Zn precursor ratio from 0.3 to 3.0, the successive redshift in absorption spectra is observed, and the corresponding Ag^+^ emission is also redshifted from 506 to 538 nm (Fig. 5c). The PL intensity, however, does not continuously enhances with increase of the In/Zn ratio but presents an optimal value at 0.7. The observed redshift of the Ag dopant emission associated with the increase of content of indium should be ascribed to the decrease in band gap of host NCs, because the proportion of low band gap In_2_S_3_ in Zn-In-S NCs increases. As a result, Zn-In-S:Ag d-NCs with tunable emission colors from 458–603 nm can be achieved in the present work by carefully controlling the reaction time, reaction temperature, Ag concentration, and the molar ratio of In to Zn (Fig. 6a).

In Zn-In-S:Ag NCs, the recombination of the exciton generated from Zn-In-S host NCs contains two possible radiative processes. One involves the transfer of Ag (I) state electrons to the valence holes, and the other one occurs with the transfer of conduction electrons to the holes trapped Ag (I) state. As to semiconductor NCs, the band gap changed by the movement of conduction band is much easier than the valence band due to the small effective mass of the electron compare to the hole^53^,^54^. So, as the band gap of NCs varies with a shift in conduction band, the recombination process occurs through the latter process, which results in a wide tunable dopant emission and rules out the radiative recombination possibility of the Ag (I) state to the valence hole. It corresponds well with our experimental results. On the basis of the above analysis, a possible mechanism schematic of exciton recombination for Zn-In-S:Ag NCs is shown in Fig. 6b. The energy level of Ag (I) state in the band gap is calculated according to the dopant emission and is found closer to the valence band for our host NCs. This result agrees well with previous investigations on the photorecombination process in Ag^+^ doped semiconductor NCs^40^,^48^.

**Figure 7:**
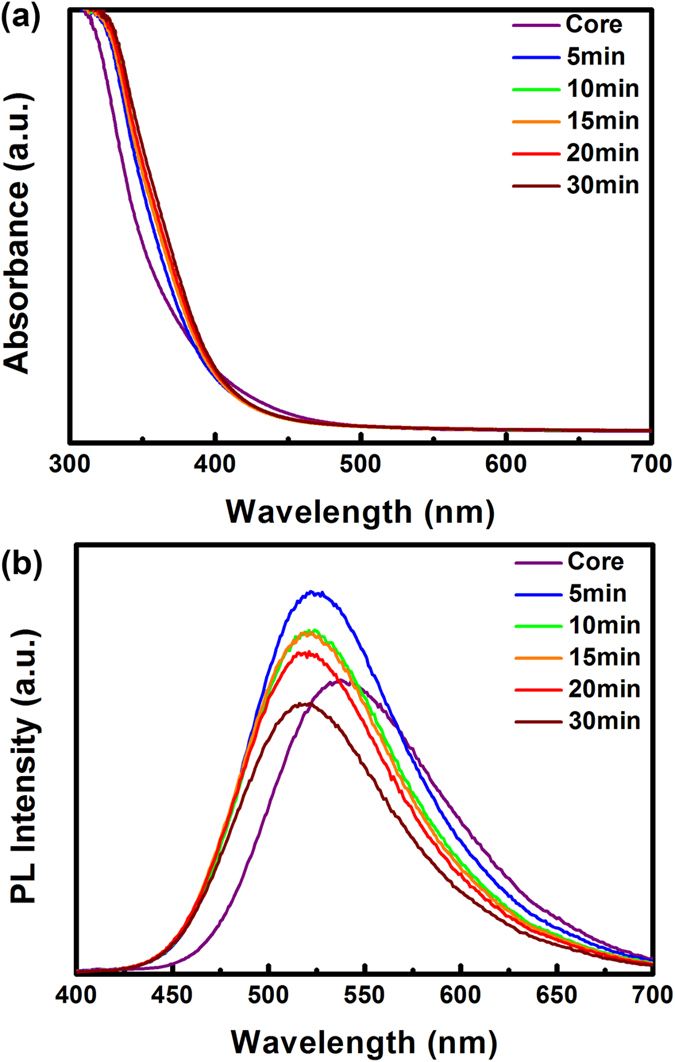
The UV/vis absorbance (**a**) and PL spectra (**b**) of Zn-In-S:Ag/ZnS core/shell d-NCs prepared at 220 °C for different reaction time.

After growing a ZnS shell around Zn-In-S:Ag d-NCs, the PL intensity is further improved owing to the evident removal of surface trap states. The PLQY of Zn-In-S:Ag/ZnS core/shell d-NCs reaches a value as high as 43.5%, which is triple that of previously reported Zn-In-Se:Ag NCs^40^. Simultaneously, an abnormal blueshift from 540–526 nm in the peak position is observed, as shown in Fig. 7b. It reveals that a small amount of zinc ions diffuse into the Zn-In-S cores and partially replace In^3+^ ions, which enlarges the band gap of NCs. This observation is well consistent with those of CuInS_2_/ZnS and AgInS_2_/ZnS core/shell NCs in the literatures^51^,^55^,^56^,^57^. Furthermore, when the Zn-In-S:Ag/ZnS NCs in chloroform are transferred into an aqueous solution through ligand exchange by 3-Mercaptopropionic acid (MPA) for applications such as cell imaging, both the position and shape of the absorption spectra as well as the body color of samples do not undergo any obvious variations. Besides this, the PL intensity of the resultant water-soluble d-NCs is only slightly decreased (~14.5%), when compared to that of the initial oil-soluble sample, as shown in Figure S2. It thus suggests that, no matter what solvents are used, the as-prepared Zn-In-S:Ag/ZnS d-NCs exhibit good PL stability because no oxidation of Ag^+^ occurs during the mercapto-ligand. Figure S3 shows the FTIR spectra of d-NCs before and after ligand exchange. Obviously, d-NCs capped by oleylamine/1-Dodecanethoil (OAm/DDT) exhibit the strong ν_as_(-CH_2_) and ν_s_(-CH_2_) asymmetric stretching vibration at 2922 cm^−1^ and 2856 cm^−1^ from OAm. After ligand exchange by MPA, the absorption band at 1698 cm^−1^ is enhanced sharply, which can be assigned to C = O stretching vibration from MPA. In contrast, the absorption bands coming from OAm almost disappear. Thus, it can be concluded that the surface of d-NCs have been successfully capped by MPA after ligand exchange.

To apply d-NCs in bio-imaging, they must have good stability and low toxicity. Therefore, the photostability of water-soluble Zn-In-S:Ag/ZnS d-NCs was investigated (Figure S4). It is clearly observed that the PL intensity of water-soluble Zn-In-S:Ag/ZnS d-NCs shows an abnormal increase, and it is about 1.25 times higher than that of the initial one after it was continuously irradiated under 365 nm for 5 h by a 6 W UV lamp. This enhancement is due to the surface oxidization, which is known for the NCs surface passivation^58^. The improved PL intensity under UV irradiations indicates high photostability of the d-NCs. To evaluate the cytotoxicity of Zn-In-S:Ag/ZnS d-NCs, KB cells were cultured with the as-prepared d-NCs and the viability was measured using the CCK-8 assay, as shown in Figure S5. Here it is noteworthy that red-emitting CdS:Cu^+^ -MPA NCs cause a 5% reduction in cell viability with a concentration of 1.5 μg mL^−1^ after 1 h exposure time^6^. In contrast, the current Zn-In-S:Ag/ZnS d-NCs present a very low toxicity, and the relative viability of cells maintains at 87% even at high dose (20 μg mL^−1^) and long incubation time (24 h). It is therefore quite safe for *in vitro* and *in vivo* applications. When it is used in the cell imaging with a relatively low concentration of 10 μg mL^−1^, the average cell viability of the d-NCs is largely improved and reaches 97% due to the absence of any toxic heavy metal ions that usually induce oxidative stress in cells.

**Figure 8:**
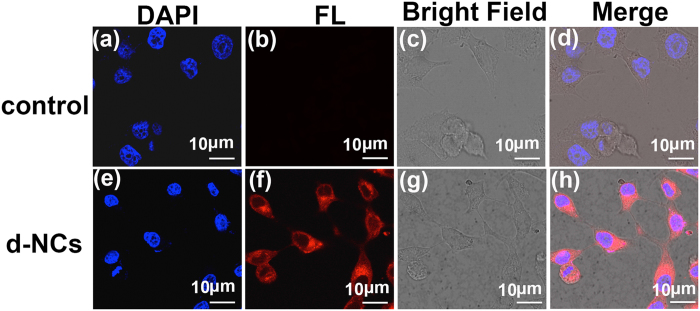
The fluorescence images (**a**,**e**) of live KB cells nuclei stained by DAPI for 30 min. Fluorescence images (**b**,**f**), bright field (**c**,**g**), and merge images (**d**,**h**) of KB cells incubated for 4 h without and with 10 μg/mL Zn-In-S:Ag/ZnS d-NCs, respectively.

**Figure 9:**
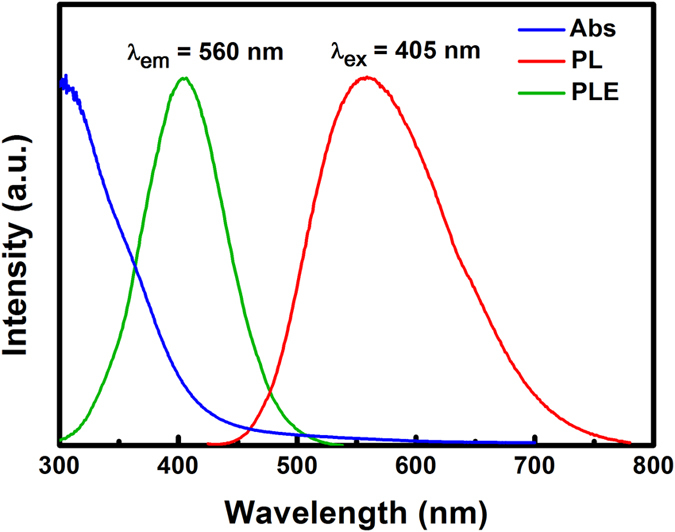
The UV/vis absorbance, PLE, and PL spectra of Zn-In-S:Ag/ZnS d-NCs dispersed in water for bio-imaging.

The potential application of the as-prepared Zn-In-S:Ag/ZnS d-NCs as bio-imaging agents was investigated in this work. The fluorescence images of living KB cells labelled with Zn-In-S:Ag/ZnS d-NCs in the aqueous solution directly are given in Fig. 8. Live KB cells were incubated for 4 h with Zn-In-S:Ag/ZnS d-NCs. The red fluorescence emission from the KB cells excited by 405 nm laser corresponds well with the emission spectrum of the d-NCs in Fig. 9, exhibiting a definite signal that the KB cells have been stained with Zn-In-S:Ag/ZnS d-NCs probe. Moreover, the image of the cells is distinct. Without d-NCs, no red fluorescence is observed for KB cells. From the DAPI, bright field and merged images, it can be seen that the shape of the cells is still spindle-like after being labelled with Zn-In-S:Ag/ZnS d-NCs, which suggests that the cells are live as before. All these phenomena demonstrate that the as-prepared Zn-In-S:Ag/ZnS d-NCs have novel biocompatibility, high stability, and low toxicity, which can serve as a potential fluorescence probe instead of Cd-containing NCs and organic dyes in the bio-imaging field.

## Conclusion

In summary, we have synthesized Cd-free Zn-In-S:Ag d-NCs with tunable emissions from 458–603 nm by a single-step noninjection method. The optical properties can be tailored by controlling the reaction time, reaction temperature, Ag^+^ concentration, and the molar ratio of In to Zn. After growing a ZnS shell, the highest PLQY of 43.5% can be achieved for Zn-In-S:Ag/ZnS d-NCs, which is triple that of previously reported Zn-In-Se:Ag NCs. Meanwhile, it exhibits high photostability and very low toxicity. Finally, the core/shell d-NCs can be easily transferred to an aqueous phase and still maintain excellent fluorescence for identifying living KB cells as bio-imaging agents.

## Method

### Chemicals

Zinc acetate (Zn(Ac)_2_, 99.5%), silver nitrate (AgNO_3_, 99.8%), sulfur powder (S, 99.99%), 1-dodecanethiol (DDT, 98.0%), methyl alcohol (analytical regent), and chloroform (analytical regent) were purchased from Sinopharm Chemical Reagent Co., Ltd. Indium acetate (In(Ac)_3_, 99.99%) and oleylamine (OAm, 70%) were purchased from Sigma-Aldrich Co. LLC. All regents were used as received without further experimental purification.

### Synthesis of Zn-In-S:Ag and Zn-In-S:Ag/ZnS d-NCs

Typically, 0.04 mmol AgNO_3_, 0.2 mmol Zn(Ac)_2_, 0.2 mmol In(Ac)_3_, 1.6 mmol S powder, 4 mL DDT, and 6 mL OAm were loaded into a 50 mL flask which was heated to 180 °C for 20 min under N2 atmosphere. Then, the mixture was cooled to 60 °C, and 5 mL of chloroform was added. The as-prepared Zn-In-S:Ag d-NCs were precipitated by adding methanol into the toluene solution, purified by repeating centrifugation, and re-dispersed in chloroform. Deposition of the ZnS shell around the Zn-In-S:Ag core NCs was carried out in the crude Zn-In-S:Ag reaction mixture solution. First, when the reaction of mixture was cooled to 100 °C, the Zn precursor (obtained by dissolving 0.4 mmol of Zn(OAc)_2_ in 0.1 mL of OAm and 0.9 mL of ODE) was loaded into the mixture. Second, the reaction system was heated to 220 °C for 15 min to allow the overgrowth the ZnS shell around the preformed Zn-In-S:Ag core NCs. Third, the purification of Zn-In-S:Ag/ZnS core/shell d-NCs was similar to that of Zn-In-S:Ag d-NCs as described above.

### Surface ligand exchange with MPA

The ligand exchange reaction was performed according to previously reported method^35^,^52^. First, the pH of 0.2 M MPA methanol solution was adjusted to 8 with tetramethylammonium hydroxide pentahydrate (TMAH). Then, 20 mL d-NCs chloroform solution (1 μM/L) and 20 mL MPA methanol solution were loaded into a vessel with strong stirring under N_2_ for 2 h at room temperature. Finally, excess TMAH and MPA were removed by centrifugation and d-NCs wrapped by MPA were re-dispersed in water for further characterization and application.

### Characterization

The absorption spectra of as-prepared QDs were measured by using a UV/vis spectrophotometer (Hitachi U-3900). Photoluminescence (PL) spectra were measured by using a fluorescence spectrophotometer (Horiba JobinYvon, FluoroMax-4). The Photoluminescence quantum yield (PLQY) was calculated by the following equation 1:


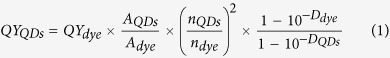


where QY, D, A, and n are the quantum yield, the optical density, the integrated area of PL spectrum, and the refractive index, respectively. The optical density at the first absorption peak of both sample and dye was kept in the range of 0.05–0.1 for avoiding reabsorption. In this paper, the dye of Rhodamine 6 G was used as a standard sample and its QY_dye_ = 95.0%. The phase evolution and morphology were characterized by a M21XVHF2Z (Mac Science Co. Ltd) X-ray diffractometer with Cu-Kα radiation (λ = 1.5406 Å, V = 40 kV, and I = 40 mA) and a JEM-2100 F transmission electron microscope (TEM), respectively. X-ray photoelectron spectroscopy (XPS) data were obtained by a multifunction imaging photoelectron spectromenter (Thermo ESCALAB 250XI) with monochromatic Al-Kα radiation (1486.6 eV). Fourier transform infrared spectra (FTIR) in the region from 4000–500 cm^−1^ were recorded on a Nicolet Nexus 600 FTIR spectroscope (Nicolet Instrument Co., USA).

### Cytotoxicity of Zn-In-S:Ag/ZnS d-NCs

To investigate the cytotoxicity of the Zn-In-S:Ag/ZnS NCs, we carried out the CCK-8 cell viability assay. The KB cells were first seeded in a 96-well plate for 24 h, and then treated with various concentration of NCs (0–20 μg/mL) in serum-containing media. After another 24 h incubation, the cells were carefully washed with PBS, then fresh medium containing CCK-8 was added into each well, and the cells were further incubated for 1 h. Finally, the relative viability of cells was assessed by measuring the absorbance of the solution at 450 nm using a microplate reader (Infinite M200, Tecan). All experiments were carried out in triplicate.

### Cell culture and cell imaging

KB cells were maintained in DMEM medium with 10% calf serum, 100 units/mL penicillin, 10 μg/mL streptomycin and 100 μg/mL neomycin in a humidified standard incubator with a 5% CO_2_ atmosphere at 37 °C. The KB cells were incubated adherently to get a suitable confluence, and stained with Zn-In-S:Ag/ZnS NCs (10 μg/mL) stick solution at 37 °C for 4 h. After washing the cells with PBS buffer, their fluorescence microscope photographs were taken by a laser scanning confocal microscope system. A 405 nm laser was used to excite cellular NCs in passing flow cell and the FL-2 channel (580–630 nm) was used to detect emissions from NCs in cells.

## Additional Information

**How to cite this article**: Xuan, T.-T. *et al.* Facile Synthesis of Cadmium-Free Zn-In-S:Ag/ZnS Nanocrystals for Bio-Imaging. *Sci. Rep.* **6**, 24459; doi: 10.1038/srep24459 (2016).

## Supplementary Material

Supplementary Information
