# Supplementary material for: Facile Synthesis of Cadmium-Free Zn-In-S:Ag/ZnS Nanocrystals for Bio-Imaging
Source: Sci Rep. 2016 Apr 14;6:24459. doi: 10.1038/srep24459 (PMC4830992; doi:10.1038/srep24459)
Supplement: Supplementary Information [file srep24459-s1.pdf]

Supporting Information

Facile Synthesis of Cadmium-Free Zn-In-S:Ag/ZnS Nanocrystals for Bio-Imaging

*Tong-Tong Xuan, Jia-Qing Liu, Cai-Yan Yu, Rong-Jun Xie, and Hui-Li Li **

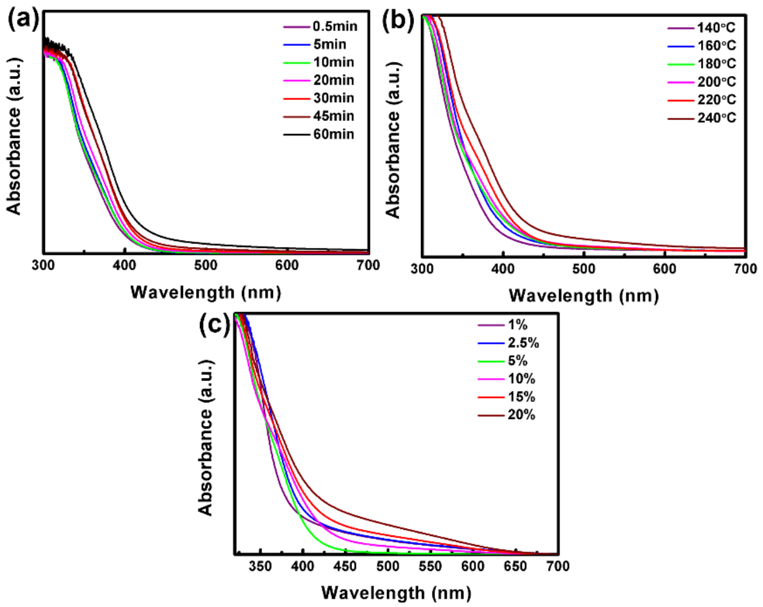

Figure. S1 The UV/vis absorption spectra of Zn-In-S:Ag d-NCs synthesized at 180 ℃ for different reaction time (a), at different reaction temperature for 20 min (b), or at 180 ℃ for 20 min with different Ag doping concentrations.

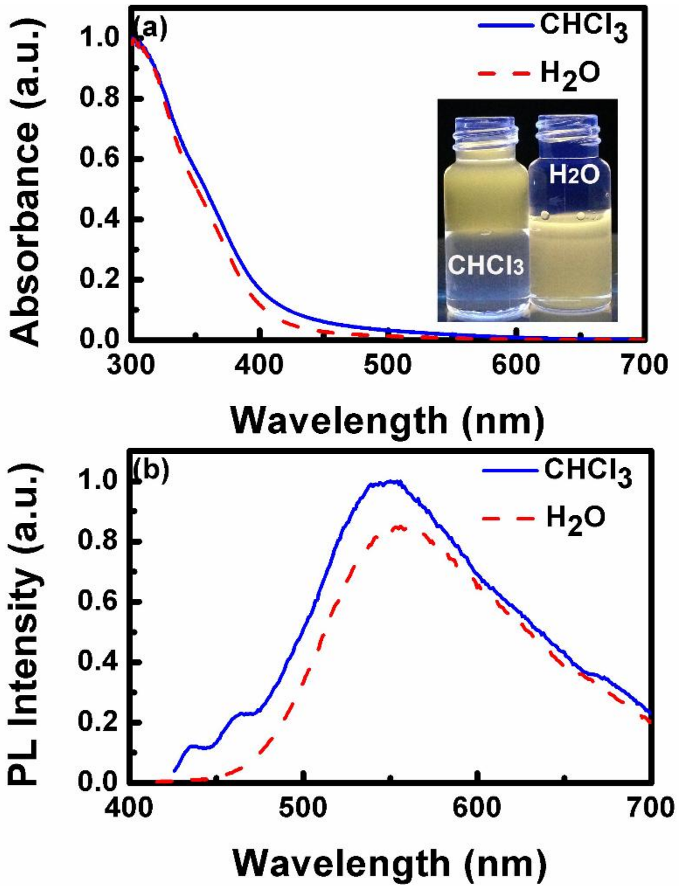

Figure. S2 The UV/vis absorption (a) and PL spectra (b) of Zn-In-S:Ag/ZnS d-NCs before ($CHCl_3$ solvent) and after (aqueous solution) phase transfer by MPA. The inset gives photographs of d-NCs in $CHCl_3$ and aqueous solution, respectively, under 365 nm UV light.

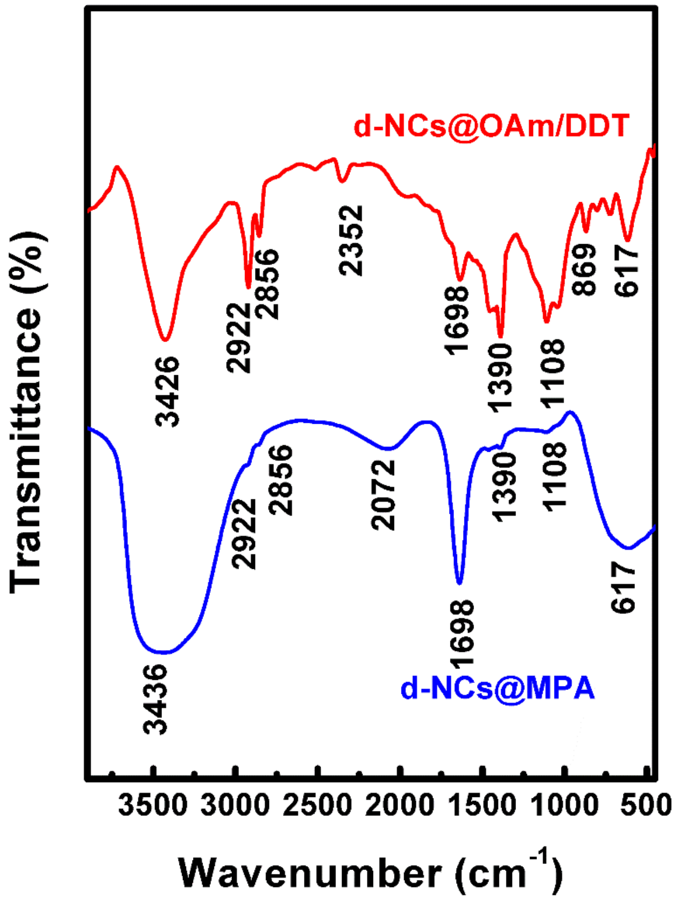

Figure. S3 FTIR spectra of Zn-In-S:Ag/ZnS d-NCs before and after ligand exchange.

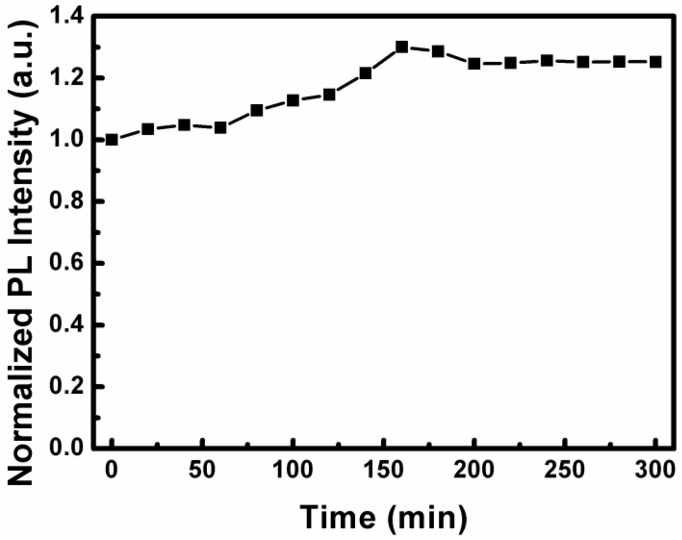

Figure. S4 Photostability of water-soluble Zn-In-S:Ag/ZnS d-NCs under 365 nm UV illumination.

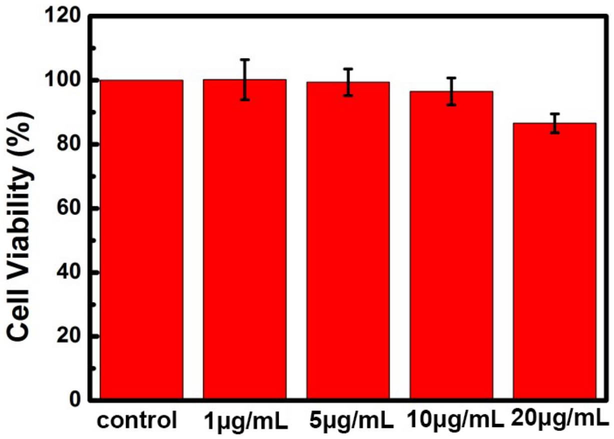

Figure. S5 The viability of KB cells in vitro measured by CCK-8 assay. The KB cells were incubated for 24 h with different concentration of Zn-In-S:Ag/ZnS d-NCs.
